# Intersecting Pathologies: Polycythemia Vera and Recurrent Infections in an Elderly Patient

**DOI:** 10.7759/cureus.80029

**Published:** 2025-03-04

**Authors:** Prince Saha, Emre Sahin, Nirupam Nadella, Adil M Siddiqui, Lokesh Edara

**Affiliations:** 1 Department of General Medicine, Dr. D. Y. Patil Medical College, Hospital & Research Centre, Dr. D. Y. Patil Vidyapeeth (Deemed to be University), Pune, IND; 2 Department of Internal Medicine, Hamidiye International School of Medicine, University of Health Sciences, Istanbul, TUR; 3 Department of Internal Medicine, Western Michigan University, Kalamazoo, USA

**Keywords:** intersecting pathologies, multidisciplinary management, myeloproliferative neoplasm, polycythemia vera, recurrent infections

## Abstract

This case report highlights the diagnostic and therapeutic challenges encountered in the management of a 95-year-old female patient with polycythemia vera (PV) complicated by recurrent infections. The patient presented with significant leukocytosis, thrombocytosis, and concurrent infections, including a urinary tract infection and influenza B. PV, a myeloproliferative neoplasm, is characterized by increased production of red blood cells and often involves heightened activation of myeloid and megakaryocytic lineages, resulting in leukocytosis and thrombocytosis. These hematological abnormalities, coupled with underlying immune dysregulation, predispose patients to recurrent and severe infections.

The patient's condition was further complicated by resistance to initial antibiotic therapy, necessitating adjustments in treatment. Despite resolving the infections, persistent leukocytosis raised concerns about the interplay between inflammatory, infectious, and neoplastic processes. This case underscores the complexities of managing patients with PV who exhibit overlapping hematological abnormalities and recurrent infections. It emphasizes the need for a multidisciplinary approach involving hematologists and infectious disease specialists to optimize care. The report also discusses the importance of preventive strategies, such as vaccinations, in reducing the risk of infections in high-risk populations, particularly those with myeloproliferative disorders.

## Introduction

Polycythemia vera (PV) is a pathological condition characterized by the excessive production of red blood cells (RBCs), leading to an increase in the overall mass of RBCs in the body [1]. Frequently, the myeloid and megakaryocytic lineages are simultaneously activated, resulting in the heightened production of white blood cells and platelets [1]. The present knowledge of pathophysiology concerns heightened responsiveness to growth factors due to an anomalous hematopoietic cell clone. Signs and symptoms such as headache, dizziness, claudication, and thrombosis can be attributed to elevated blood viscosity [1].

Leukocytosis, usually referred to as an increased white blood cell count, is a frequently observed laboratory abnormality in both inpatient acute care settings and outpatient practices [2]. It has multiple causes, such as infection, inflammation, and malignancy, that practitioners from all disciplines should be knowledgeable about [2].

Thrombocytosis is a condition characterized by excessive platelet production in the body. Reactive thrombocytosis, also known as secondary thrombocytosis, occurs when an underlying ailment, such as an infection, causes it [3]. Occasionally, when there is no apparent underlying cause for a high platelet count, the illness is referred to as primary thrombocythemia, essential thrombocythemia, or PV [3]. This condition is characterized by abnormalities in the blood and bone marrow [3].

This report provides a unique case during the approach to leukocytosis and thrombocytosis, including their differential diagnosis. It also discusses the challenges faced by the interprofessional team involved in patient care when considering treatment options and their effectiveness in cases where multiple factors contribute to elevated counts.

## Case presentation

A 95-year-old Hispanic woman was transferred from an Adult Living Facility (ALF) to the emergency room on March 11, 2024, at a (secondary health care center) in Miami-Dade County, Florida. She has a past medical history of (Janus Kinase) JAK-2 positive PV treated with hydroxyurea, hyperlipidemia, hypothyroidism, dementia, hypertension, pneumonia, total hip replacement, and coronary artery disease. She came with complaints of right facial swelling, redness, and progressive generalized weakness. She was later found to have significant leukocytosis at around 30,000 WBCs per microliter and significant hematuria and pyuria suggestive of a urinary tract infection (UTI).

**Figure 1:**
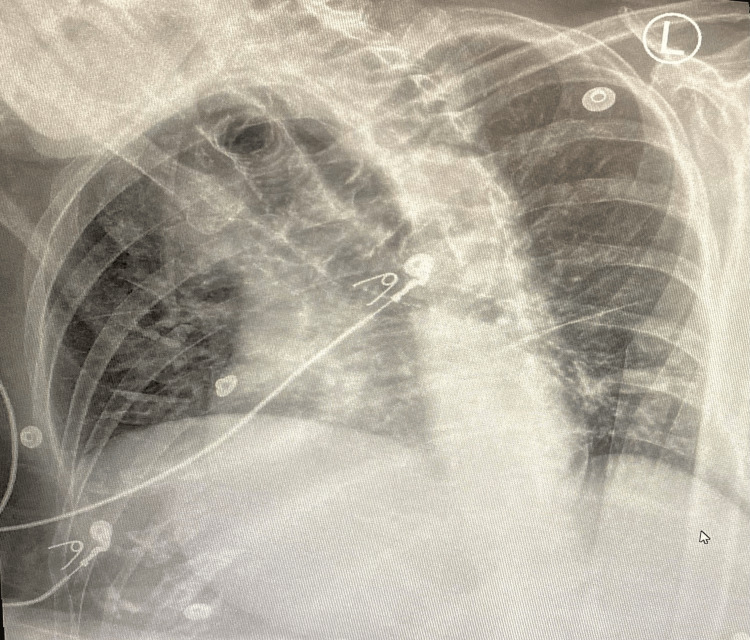
Chest X-ray of the patient The chest X-ray failed to reveal any consolidation

During the physical examination, the patient appeared frail and lethargic but not distressed, with a T-max of 97.6F. Her neurological examination revealed AAOx1, meaning she was oriented only to people. The remainder of the examination was within normal limits. Significant laboratory and radiological findings at the time of presentation are presented in Tables 1-3 and Figure 1.

**Table 1:** Hematological Parameters at Presentation

cTest	Results	Units	Normal Range
White Blood Cells (WBCs)	30.5	10^3^/mcL	5.2-10
Neutrophils% (Manual)	98	%	52-73
Lymphocytes% (Manual)	2	%	20-45
Neutrophils# (Manual)	29.89	10^3^/mcL	1.47-6.20
Lymphocytes# (Manual)	0.6	10^3^/mcL	1.3-4.2
Toxic Granulations	Slight		
Prothrombin Time (PT)	13.8	seconds	9.3-12.1
International Normalized Ratio (INR)	1.3		0.9-1.2
Activated Partial Thromboplastin Time (aPTT)	37.0	seconds	23.9-32.8
Hemoglobin	9.3	gm/dL	12.3-15.3
Hematocrit	32.7	%	36.0-45.0
Mean Corpuscular Volume (MCV)	80.0	fL	81.0-99.0
Mean Corpuscular Hemoglobin (MCH)	22.7	pg	26.0-32.0
Mean Corpuscular Hemoglobin Concentration (MCHC)	28.4	gm/dL	31.0-35.4
Red cell Distribution Width (RDW)	22.1	%	11.5-14.5
Platelet Count	571	10^3^/mcL	130-433
Blood Urea Nitrogen (BUN)	26	mg/dL	7.0-17.0
Creatinine	1.10	mg/dL	0.52-1.04
Estimated Creatinine Clearance	28	mL/min	88–128
Estimated GFR	46	mL/min/1.73m^2^	>=90
BUN/Creatinine Ratio	23.6		10:1-20:1

**Table 2:** Urinalysis Findings at Presentation

Test	Results	Units	Normal Range
Urine Color	Amber		Yellow
Urine Clarity	Cloudy		Clear
Urine pH	5.0		5.0-8.0
Urine Specific Gravity	1.018		1.000-1.035
Urine Protein	100	mg/dL	Negative
Urine Ketones	5	mg/dL	Negative
Urine Blood	Large		Negative
Urine Leukocyte Esterase	Moderate		Negative
Urine RBC	>182	/hpf	0-2
Urine WBC	90	/hpf	0-5
Urine WBC clumps	7	/hpf	0-1
Urine Squamous Epithelial Cells	2	/hpf	0-5
Urine Bacteria	Moderate	/hpf	None Seen
Urine Mucus	Occassional	/lpf	None Seen

**Table 3:** Results of Rapid Antigen Testing for Influenza and COVID-19

Test	Results	Units	Normal Range
Influenza Type A Antigen	Negative		Negative
Influenza Type B Antigen	Positive		Negative
SARS-CoV-2 Ag (Rapid)	Negative		Negative

The blood analysis showed evidence of microcytic anemia, indicating a possible iron deficit. Additionally, there was an increase in platelet count (thrombocytosis) and white blood cells (WBCs) with minor toxicity with toxic granulations (granulocytic leukocytosis). The kidney function test indicated the presence of acute kidney injury in the patient, as evidenced by elevated creatinine levels and abnormal values of BUN and BUN/creatinine ratio, as shown in Table 1. The urine routine testing revealed the presence of hematuria and pyuria, along with elevated leukocyte esterase and urine bacteria, confirming the diagnosis of a UTI, as shown in Table 2. Cultures were obtained, and based on empirical evidence, the patient was initiated on cefepime and vancomycin.

The rapid antigen testing results for influenza B came out to be positive, as shown in Table 3. A chest X-ray was subsequently ordered, but it failed to reveal any consolidation, as shown in Figure 1. The patient was appropriately started on oseltamivir therapy. The SARS-CoV-2 rapid antigen testing came out negative. Due to the patient's repeated history of UTIs, cefepime was replaced with meropenem. The vancomycin and oseltamivir treatment was appropriately modified to avoid any difficulties due to toxicity, considering the patient's advanced age and impaired renal function.

The culture ultimately cultivated Enterococcus species, specifically Enterococcus faecium. Additional bacterial susceptibility pattern testing revealed that the bacterium exhibited resistance to vancomycin. Subsequently, treatment with linezolid was initiated, and the patient's clinical condition improved significantly.

Nevertheless, despite completing the treatment, the WBC count remained high, causing uncertainty regarding whether the increased number of leukocytes resulted from an inflammatory, infectious, or malignant process occurring in the background. It is challenging to accurately diagnose the condition, as in this patient with an ongoing infection (UTI & Influenza B) and underlying myeloproliferative disorder (PV) and determine the appropriate treatment choice and duration. 

## Discussion

PV leads to excessive RBC production, often accompanied by concurrent activation of myeloid and megakaryocytic lineages, boosting WBC (leukocytosis) and platelet counts (thrombocytosis) [1]. Leukocytosis and thrombocytosis can each have their respective etiologies, complications, and further implications in a patient, and in the background of a myeloproliferative disorder, it can be challenging to pinpoint the exact pathological condition contributing to these signs, thereby putting forward a challenge to the interdisciplinary team involved in patient care [1].

A study conducted by Luque et al. discovered that individuals with myeloproliferative neoplasms (MPNs) have chronic inflammation and an imbalance in their immune system, resulting in elevated levels of pro-inflammatory cytokines [4]. MPNs are associated with alterations in immune cell populations, particularly B lymphocytes. Studies indicate that patients with MPNs often exhibit reduced numbers of B lymphocytes, which inversely correlates with the burden of the JAK2-V617F allele [5]. The implications of this observation suggest a potential compromise in immune function, which may contribute to an increased vulnerability to infections among these individuals [5]. Understanding these relationships is crucial for developing targeted therapies and improving patient management strategies in MPNs.

Romano et al., in their study, found that abnormalities in the immune system vary among patients with different driver mutations [6]. Specifically, JAK2-mutated cases showed more alterations in T-regulatory cell populations, while CALR-mutated cases exhibited dysregulations in the interferon-γ-axis [6]. Furthermore, individuals with MPNs are also more susceptible to other medical conditions, including cardiovascular and thromboembolic disease, autoimmune disease, and solid malignancies [7-9]. As a result, they may have a higher likelihood of developing infections due to the treatment of these comorbidities, hospitalization for unrelated reasons, or overall increased vulnerability. The higher risk of diseases found in MPNs may be attributed to the simultaneous presence of multiple underlying causes [7-9].

There is widespread support for immunization against pneumococci and influenza in Sweden for adults aged 65 and over and for specific risk categories (MPNs not included) [10].

However, the provision and subsidization of these vaccinations for residents may vary across different counties [10]. Since 2009, health authorities have included pneumococci immunization in the recommended routine for babies [10]. In 2014, reports indicated that 50% of the elderly received the influenza vaccine [10].

Since 2013, Sweden has offered vaccination against the reactivation of zoster to individuals who specifically request it, although this vaccination is not commonly advised [11]. The determination of appropriate preventive or prophylactic actions for individuals with MPNs cannot solely rely on observational findings [11]. However, it may be prudent to consider some vaccines, such as the varicella zoster vaccine, for specific high-risk individuals [11].

According to Landtblom et al., individuals with MPNs had a considerably greater susceptibility to severe infections when compared to a control group from the general population [11]. The absence of disparities in infection risk between untreated and treated patients in their study indicated that the heightened risk of infections is an inherent characteristic of MPNs [11]. Therefore, the risk of infection should not be a decisive factor when contemplating cytoreductive treatments like hydroxyurea or interferon-α if they are otherwise indicated [11]. It is essential to acknowledge the heightened vulnerability to infections while treating patients with MPNs and when assessing the impact of drugs, especially JAK inhibitors, on their susceptibility to infections.

## Conclusions

In conclusion, the case demonstrates the complex difficulties faced when diagnosing and treating patients with PV who also have high WBC count, high platelet count, and illnesses such as UTIs and influenza. The interaction between the underlying myeloproliferative illness and the infectious processes adds complexity to the diagnosis and therapy considerations. The discussion elucidates the immunological dysregulation exhibited in people with MPNs, which makes them more prone to chronic inflammation and infections. Moreover, the diverse irregularities in the immune system among individuals with distinct driver mutations highlight the intricacy of handling issues connected to MPNs. Emphasis is placed on the significance of preventative interventions, such as immunization against pneumococci and influenza, for high-risk persons. Nevertheless, the choice regarding preventive measures should be approached with care, considering specific patient characteristics and the existing data. Furthermore, the increased susceptibility to infections highlights the importance of thorough assessment and control of infectious consequences, particularly while undergoing cytoreductive therapies like hydroxyurea or interferon-α. The results indicate that the potential for infection should not discourage the proper utilization of these treatments when necessary. To summarize, this study highlights the significance of a multidisciplinary strategy that includes hematologists, infectious disease specialists, and other healthcare providers in effectively managing patients with PV and its related consequences. Additional studies are necessary to gain a deeper understanding of the immunological pathways that contribute to infections connected to MPNs and enhance preventive and treatment approaches for these individuals.
